# The Annual Meeting of the Poor-Law Medical Officers' Association of England and Wales

**Published:** 1914-07-11

**Authors:** 


### The Annual Meeting of the Poor-Law Medical Officers' Association of England and Wales.

The annual meeting of the Poor-Law Medical Officers' Association was held at the Town Hall, Burnley, on July 2, the President, Surg.-General Evatt, C.B., being in the chair.

An interesting paper was read by Mr. C. E. Bygrave, Clerk to the Blackburn Guardians, upon "Recent Legislation Affecting the Duties of Poor-Law Medical Officers." He said that when the National Insurance Act was passed it was anticipated that the duties of the district medical officer would be considerably lessened as the direct result of the provisions of the Act. Inquiries made in the Blackburn Union showed that those anticipations had not been realised. The relieving officers reported that on the whole they found no appreciable difference in the issue of medical orders, and there appeared to be no diminution in the need for institutional medical treatment. A matter of interest to the workhouse medical officers was their position in the matter of applications to them by insured persons in the workhouse infirmary for certificates to enable them to recover from their approved societies the benefits due to them whilst in the infirmary. The workhouse medical officer was under no legal obligation to fill in the certificates of the Approved Societies. He could only be required to give a certificate of the nature of the illness for which he was attending an inmate. The training of probationers in the Poor-Law infirmary was the subject of much discussion just now, and there appeared to be a consensus of opinion that a standard curriculum and one central examination were required. If a scheme of this nature were adopted, it would mean an increase in the work of the infirmary medical officer. It seemed to be the general opinion that the recent Poor-Law Institutions Order had considerably increased the work of most workhouse medical officers. He was not convinced that this was the case. The work of keeping the new record papers should be set off against the work entailed in keeping the medical relief register, abolished under the new Order. The question of the remuneration of workhouse medical officers for attending and giving evidence at inquests required putting upon a more satisfactory basis. In some unions fees were paid, in others they were not, and the decision seemed to depend entirely upon the way in which individual coroners construed the Coroners Act of 1887. The proposal to substitute whole-time for part-time district medical officers was not likely to mature, at any rate in rural districts. Even if the positions of medical officer of health and district medical officer were combined, the salary offered would not be likely to attract competent men.

Dr. Major Greenwood, Senior District Medical Officer for Shoreditch, then read a paper on "The Future of the Poor Law and the Poor-Law Medical Service." Dr. Greenwood said that any attempt to foretell the future must be based upon the history of the past. Under the feudal system the responsibility for the relief of the poor was thrown upon the overlord and the great religious houses. The sparseness of the population rendered the problem a comparatively easy one. Charity was the dominant principle upon which relief was given. The first attempt of the State to grapple with poverty was in the reign of Elizabeth. Charity had little to do with the State aid granted to the poor by our early Poor-Law legislation. The destitute were regarded as criminals, and it must not be forgotten that the evil reputation that still attached to the Poor Law was an inheritance from those days. The keystone of our present system was the Poor-Law Amendment Act of 1834. Since then there had been no direct legislation, but a large number of Local Government Board Orders had been issued, tending to amplify the provisions of the Act and to render its working more efficient and humane. Recently many enactments connected with social reform had largely trenched on the ground heretofore reserved for the Poor Law. He referred especially to the Old Age Pension Acts, the Elementary Education Acts, and the National Insurance Acts. These measures had greatly complicated the working of the Poor Law, and led to much overlapping. Not their least mischievous effect was the tendency they had to obscure the actual amount of pauperism in the country, and to grant relief without supervision by skilled officials. Public discontent with the Poor-Law system resulted in 1906 in the appointment of a Royal Commission. Unfortunately the Commissioners were not unanimous in their recommendations, and this disagreement no doubt explained in part why no attempt had been made to legislate on the subject. No doubt, however, when the present pressure of Parliamentary business was lightened, further Poor-Law legislation would follow. It was, therefore, essential that the Poor-Law service should be adequately organised so as to exercise a guiding influence. Narrow views as to the interests of the service itself ought not to dominate their counsels. As regards the indoor medical service, the Poor-Law infirmary would continue to approximate in equipment and staffing to the voluntary hospital. Central infirmaries for the treatment of the sick over large areas formed by the combination of unions would probably be established. As regards the outdoor medical service, he was strongly opposed to the creation of whole-time district medical officers acting under the medical superintendent of the local infirmary. Such officers had no security of tenure of office, and could not be so independent as the part-time medical officer. They would in most cases be junior practitioners, and the poor would not have the same confidence in them that they had in the part-time district medical officer, whose practice was not confined to one section of the community, but included both rich and poor.

* In this connection we refer our readers to the Bill drafted by the Coroners' Society, *see* p. 412 of this issue.—Ed.

